# Novel Nrf2-Inducer Prevents Mitochondrial Defects and Oxidative Stress in Friedreich’s Ataxia Models

**DOI:** 10.3389/fncel.2018.00188

**Published:** 2018-07-17

**Authors:** Rosella Abeti, Annalisa Baccaro, Noemi Esteras, Paola Giunti

**Affiliations:** ^1^Ataxia Centre, Department of Molecular Neuroscience, UCL Institute of Neurology, London, United Kingdom; ^2^Department of Molecular Neuroscience, UCL Institute of Neurology, London, United Kingdom

**Keywords:** Friedreich’s ataxia, reactive oxygen species, lipid peroxidation, mitochondrial dysfunction, human fibroblasts, nuclear factor (erythroid-derived 2)-like 2

## Abstract

Friedreich’s Ataxia (FRDA) is an autosomal recessive neurodegenerative disorder, affecting dorsal root ganglia (DRG), cerebellar dentate nuclei and heart. It is caused by a GAA repeat expansion mutation within the frataxin gene (*FXN*). This impedes *FXN* transcription resulting in a progressive decrease of the mitochondrial protein, frataxin. Increased oxidative stress leading to a chronic depletion of endogenous antioxidants affects the survival of the cells and causes neurodegeneration. In particular, cerebellar granule neurons (CGNs) show a significant increase of reactive oxygen species (ROS), lipid peroxidation and lower level of reduced glutathione (GSH). In FRDA, one of the major pathways of oxidant scavengers, the Nrf2 antioxidant pathway, is defective. Previous studies on FRDA-like CGNs showed that the reduced level of frataxin and the oxidative stress induce mitochondrial impairments. By triggering the Nrf2 endogenous pathway pharmacologically we determined whether this could promote mitochondrial fitness and counteract oxidative stress. In this work, we sought to investigate the beneficial effect of a promising Nrf2-inducer, omaveloxolone (omav), in CGNs from two FRDA mouse models, KIKO and YG8R, and human fibroblasts from patients. We found that CGNs from both KIKO and YG8R presented Complex I deficiency and that omav was able to restore substrate availability and Complex I activity. This was also confirmed in human primary fibroblasts from FRDA patients. Although fibroblasts are not the major tissue affected, we found that they show significant differences recapitulating the disease; this is therefore an important tool to investigate patients’ pathophysiology. Interestingly, we found that patient fibroblasts had an increased level of endogenous lipid peroxidation and mitochondrial ROS (mROS), and lower GSH at rest. Omav was able to reverse this phenotype, protecting the cells against oxidative stress. By stimulating the cells with hydrogen peroxide (H_2_O_2_) and looking for potential mitochondrial pathophysiology, we found that fibroblasts could not maintain their mitochondrial membrane potential (ΔΨ_m_). Remarkably, omav was protective to mitochondrial depolarization, promoting mitochondrial respiration and preventing cell death. Our results show that omav promotes Complex I activity and protect cells from oxidative stress. Omav could, therefore, be used as a novel therapeutic drug to ameliorate the pathophysiology of FRDA.

## Introduction

Friedreich’s Ataxia (FRDA) is a rare autosomal recessive disorder. Patients develop dorsal root ganglia (DRG) and dentate nucleate atrophy and cardiomyopathy (Parkinson et al., 2013). The disease-causing mutation is harbored within the frataxin gene (FXN), impeding the transcription of the gene and resulting in a decrease of the final product, the mitochondrial protein, frataxin (Campuzano et al., 1996). Frataxin is crucial for the biogenesis of iron sulfur clusters (ISCs; Radisky et al., 1999; Lesuisse et al., 2003; Martelli and Puccio, 2014). Impairments of ISC biogenesis result in accumulation of iron free radicals and lack of ISCs to serve aconitase and complex I, II and III of the electron transport chain (ETC) within the mitochondria (Al-Mahdawi et al., 2006; Condò et al., 2010). Mitochondria are the sites where the cell produces energy and generates reactive oxygen species (ROS; Nickel et al., 2014). The pathogenic mechanism triggered by the reduced production of frataxin leads to the generation of oxidative stress, mitochondrial energy imbalance and an increase in lipid peroxidation. This has been shown in cerebellar granule neurons (CGNs), and mouse fibroblasts (Abeti et al., 2015, 2016). In different models, fibroblasts from patients showed a marked sensitivity to pro-oxidant agents (Paupe et al., 2009; Shan et al., 2013). Indeed, human fibroblasts have shown a defective Nuclear factor (erythroid-derived 2)-like 2 (Nrf2) pathway, due to Nrf2 failure to translocate from cytosol to nucleus (Paupe et al., 2009). The Nrf2 pathway is one of the major antioxidant and ROS scavenging pathways which defends the cells against oxidative stress and regulates mitochondrial metabolism and function (Esteras et al., 2016). In primary fibroblasts from a FRDA-like mouse model, we found that Nrf2 inducers were protective against the increase in lipid peroxidation, mitochondrial energy imbalance and cell death (Abeti et al., 2015). Amongst the therapeutic strategies applied to FRDA, over the years, antioxidants have been shown to have a positive outcome (Cooper and Schapira, 2007; Cooper et al., 2008). In this work, we have investigated the beneficial effect on oxidative stress and mitochondrial function of omaveloxolone (omav) which specifically triggers the Nrf2 pathway (Creelan et al., 2017). The turnover of Nrf2 is regulated through ubiquitination and proteasomal degradation. Nrf2 is one of the main components driving the transcription of antioxidant responsive elements (ARE) in the presence of high levels of oxidation in the cells. The proteins involved in Nrf2 regulation are Kelch-like ECH-associated protein 1 (Keap1), which acts as negative regulator of Nrf2 (Itoh et al., 1999); glycogen synthase kinase 3 (GSK3), which phosphorylates Nrf2 and drives it towards degradation via β-transducin repeats-containing protein (β-TrCP-Cul1-based ubiquitin ligase); and E3 ubiquitin ligase synoviolin (Hrd1), which takes part in protein degradation in the endoplasmic reticulum (Wu et al., 2014). Omav is a new Nrf2 activator that prevents the ubiquitination of Nrf2, increasing its levels (Reisman et al., 2014a; Holmström et al., 2016). In cellular models of FRDA, the Nrf2 pathway seems to be suppressed and Nrf2 fails to undergo nuclear translocation in cellular models of FRDA (Paupe et al., 2009). Moreover, Nrf2 target proteins are also downregulated in FRDA. We now investigated the effect of omav in CGNs from two mouse models, KIKO and YG8R, and FRDA human fibroblasts. We found that omav was able to protect the cells from a marked Complex I inhibition as measured by nicotinamide adenine dinucleotide (NADH) autofluorescence and was able to increase the mitochondrial NADH pool and activate respiration across models. Moreover, omav was able to counteract oxidative stress, as detected by lipid peroxidation, mitochondrial ROS (mROS) and reduced glutathione (GSH) levels. By looking at the mitochondrial membrane potential (ΔΨ_m_), a marker of mitochondrial health, we found that FRDA cells pre-treated with omav were able to maintain their ΔΨ_m_, indicating its effect on oxidative stress had also a positive impact on mitochondrial function. Finally, we found that this compound was protective against hydrogen peroxide (H_2_O_2_)-induced cell death in FRDA fibroblasts. This shows that omav could potentially be used as a novel therapeutic drug in FRDA, to ameliorate the pathophysiology of this impairing disorder.

## Materials and Methods

### Cerebellar Granule Neurons

CGNs from KIKO (*mFxn^–/+230 GAA^*; From Jackson Laboratory; Marmolino et al., 2010) and YG8R mice (*mFxn*^−/−^ and *hFXN^−/+82 and 190GAA^*; From Jackson Laboratory; Al-Mahdawi et al., 2006) and their controls, respectively WT and Y47R (*mFxn*^−/−^ and *hFXN*^–/+9GAA^; From Jackson Laboratory) were isolated with the method described in Abeti et al. (2016) with few modifications. In brief, cerebella were isolated and homogenized and dissociated with 0.25% trypsin-EDTA (Sigma Aldrich). CGNs were plated on, pre-coated with Poly-D-lysine (1 mg/ml), glass coverslips. Cells were maintained in Neurobasal with 2% of B27 (Invitrogen) and 2 mM L-glutamine, 1% of penicillin/streptomycin (Sigma Aldrich) and 25 mM KCL (to keep the CGNs slightly depolarized; Scorziello et al., 1996). To avoid glial proliferation 10 μM AraC was added after 24 h from the plating. CGNs were used 7 days after plating. Cells were maintained in a humidified incubator at 37°C and at 5% CO_2_. Prior experiments cells were treated 24 h with 50 nM omav.

### Primary Human Fibroblasts

Primary Human Fibroblasts were extracted from skin biopsy samples from patients and control subjects (Table 1, Control, Carrier, Patient 1 and 2). Fibroblasts were maintained in Dulbecco’s Eagle Modified (DMEM) Glutamax (Invitrogen), with 10% Foetal Bovine Serum (FBS; Invitrogen). Cells were harvested with 0.25% trypsin-EDTA (Sigma Aldrich), then inactivated with complete media and plated on 25 mm glass coverslips or directly into a six multi-wells plate, according to the type of experiment.

**Table 1:** Patients GAA repeats.

	GAA repeats
Control	N/A
Carrier	N/A
Patient 1	850/1186
Patient 2	1000/1000

*Control was unaffected; Carrier had one copy of the mutation. Patients GAA repeats are indicated in the table*.

### Lipid Peroxidation Measurements

Cells were loaded with 10 μM C11 BODIPY (581/591) for 10 min, which measures lipid peroxidation in the cell. The dye was excited at 488 nm (oxidized form) and 561 nm (unoxidized form; Abeti et al., 2015, 2016), detected with 710 Zeiss Confocal VIS CLSM equipped with a META detection system and a 40× oil immersion objective. The ratio of 488/561 nm was analyzed and the rates were then calculated in ArbU/min.

### Mitochondrial ROS

Cells were loaded with 1 μM MitoTracker Red CM-H_2_Xros (Thermo Scientific) incubated for 20 min before the beginning of the experiments, and then imaged the increase of fluorescence over time using 561 nm excitation and long pass filter. MitoTracker Red CM-H_2_Xros is a reduced, non-fluorescent dye which fluoresces upon oxidation and enters the mitochondria according to the membrane potential. Confocal images were obtained using a Zeiss 710 VIS CLSM equipped with a META detection system and a 40× oil immersion objective.

### Measurement of ΔΨ_m_

Tetramethyl rhodamine, methyl ester (TMRM) is a cationic, cell-permeant fluorescent dye readily sequestered by healthy mitochondria. Basal mitochondrial membrane potential was measured in the non-quench mode, loading the cells with 25 nM TMRM for 40 min, z-stacks were acquired with a 710 Zeiss Confocal microscope. To measure small differences in the maintenance of ΔΨ_m_ we used a de-quenching mode. Cells were incubated for 20 min with 1 μM TMRM. Depolarization, after oligomycin (2 μg/mL) administration, was detected as an increase of ΔΨ_m_. At the end of each experiment, 1 μM Carbonyl cyanide-*4*-(trifluoromethoxy)phenylhydrazone (FCCP) was applied. FCCP is protonophore that dissipates the potential completely. TMRM was visualized using an excitation wavelength of 561 nm with a long pass filter using a Zeiss 710 VIS CLSM equipped with a META detection system and a 40× oil immersion objective.

### Glutathione Measurements

Reduced Glutathione (GSH) was measured with monochlorobimane (MCB), a non-fluorescent compound that becomes fluorescent when binds GSH. Cells were incubated with 50 μM MCB for 30 min. Confocal images (z-stacks) were acquired for quantitation using a Zeiss 710 VIS CLSM equipped with a META detection system and a 40× oil immersion objective.

### NADH Pool and Redox State

The autofluorescence of NADH and NADPH (which can be referred to NAD(P)H) in fibroblast cultures was imaged on a cooled charge-coupled device (CCD) camera (Hamamatsu, Orca ER). The blue autofluorescence, emitted by the pyridine nucleotides NADH and NADPH in their reduced form, was excited with a 360 nm and emission was collected using a 455 nm filter. Confocal images were acquired using a Zeiss 510 UV LSM system and a 63× water immersion objective. The application of mitochondrial uncoupler 1 μM FCCP maximizes the rate of respiration and oxidizes the mitochondrial NADH pool in cells, resulting in a decrease of detected fluorescence (minimum = 0% for NADH). The subsequent application of the Complex IV inhibitor, 1 mM NaCN, suppresses respiration preventing NADH oxidation and allowing the NADH pool to be regenerated (maximum = 100% for NADH). The final formula used to normalize the NADH autofluorescence measurement was: ΔF − Ffccp/ΔFNaCN − Ffccp (Abeti et al., 2011). Quantitative analysis of the images obtained was done cell by cell using the AQM Andor software, the average was taken from *n* > 3 independent experiments for each condition.

### Cell Death

Cells were treated 24 h with 50 nM omav, 2 h with 1 mM H_2_O_2_. Prior imaging, cells were incubated with propidium iodide (PI; 10 μM) and 300 nM DAPI for 15 min, washed 3× with PBS 1× and analyzed using a cooled CCD camera. DAPI stains all nuclei while PI stains only cells with a disrupted plasma membrane. Dead fibroblasts (PI positive), were counted as a fraction of the total. In each experiment, were examined five random fields and the mean is representative of three independent experiments for each condition.

### Statistical Analysis

Statistical analysis was performed with Excel, Origin 9 (Microcal Software Inc.) and GraphPad5 software (GraphPad Software, La Jolla, CA, USA). Results are expressed as means ± S.E.M or S.D.M. The ANOVA test was applied when appropriate and the point of minimum acceptable statistical significance was taken to be 0.05, and was Bonferroni corrected where required. Representative averages were taken from *n* > 3 independent experiments.

## Results

### Complex I Inhibition Present Across FRDA Models

**Figure 1:**
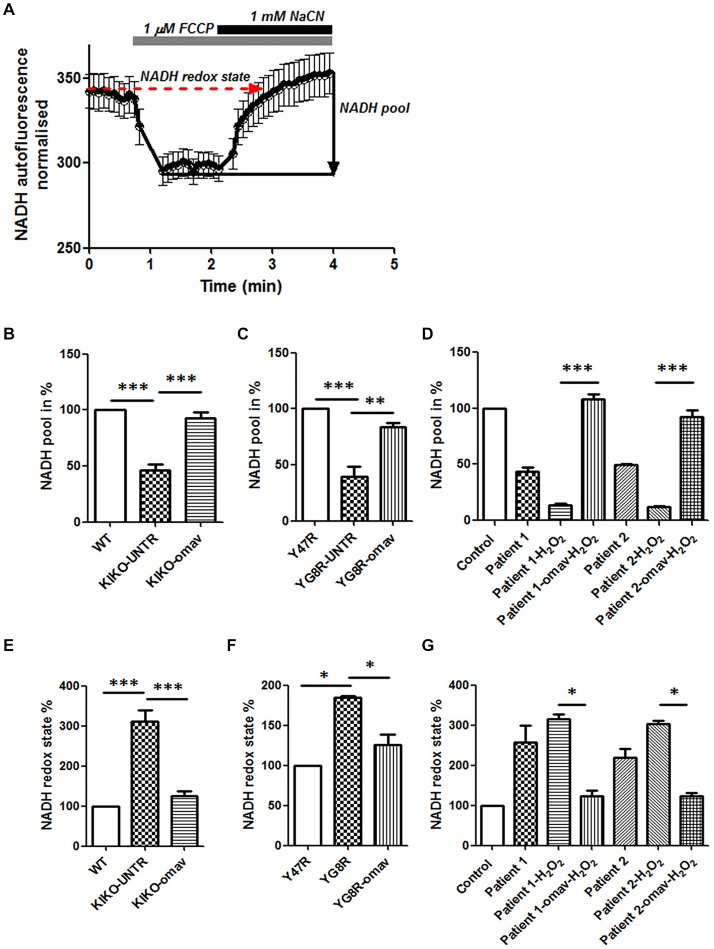
Omaveloxolone (Omav) prevents Complex I inhibition in friedreich’s ataxia (FRDA) fibroblasts and neurons. **(A)** Representative trace of nicotinamide adenine dinucleotide (NADH) autofluorescence where the basal level refers to the NADH redox state and the response to NaCN subtracting the response to carbonyl cyanide-4-(trifluoromethoxy)phenylhydrazone (FCCP) represents the NADH pool. **(B–D)** The histograms represent the calculation total NADH pool in KIKO, YG8R cerebellar granule neurons (CGNs) and Patients compared to their controls and without/with omav (**p < 0.005; ***p < 0.0005). **(E–G)** The histograms represent the calculation total NADH redox state in KIKO, YG8R CGNs and Patients compared to their controls and without/with omav (*p < 0.05; ***p < 0.0005).

In FRDA, the full function of the mitochondrial ETC is partially impaired as the lack of frataxin inhibits the production of ISCs which are crucial for the functional activity of Complex I, II and III. Amongst the three Complexes, Complex I requires more ISCs and is therefore more sensitive to changes in frataxin level. By measuring NADH autofluorescence, we could detect the levels of the mitochondrial NADH pool available and also the NADH redox state to prove Complex I deficiency and understand whether or not omav could restore it. Figure 1 shows a representative trace of NADH autofluorescence measurements in FRDA cells, and their response to 1 μM FCCP and 1 mM NaCN (Figure 1A; Bartolomé and Abramov, 2015). We found that the level of NADH pool in CGNs from KIKO and YG8R mice was lower compared to their controls and that 50 nM omav pre-incubated for 24 h was able to restore it (Figures 1B,C; KIKO-UNTR 46.5 ± 8.2, KIKO-omav 92.6 ± 9.8, ***p < 0.0005; YG8R-UNTR 39.8 ± 14.4, ***p < 0.0005, YG8R-omav 84 ± 5.5, **p < 0.005). Similar results were achieved in FRDA patient’s fibroblasts. Figure 1D shows the NADH pool in fibroblasts untreated and challenged with a pro-oxidant (hydrogen peroxide; 1 mM H_2_O_2_; Figure 1D; Patient 1 43.48 ± 7.8, Patient 1_H_2_O_2_ 13.33 ± 3.8, Patient 1_omav_H_2_O_2_ 107.8 ± 10.15, Patient 2 49.58 ± 1.08, Patient 2_H_2_O_2_ 12.04 ± 2.3, Patient 2_ omav_H_2_O_2_ 92.73 ± 12; ***p < 0.0005). Across FRDA models the NADH redox state was higher than controls and was recovered by pre-treating them with omav (Figures 1E–G; KIKO-UNTR 312 ± 45, KIKO-omav 124.3 ± 23, ***p < 0.0005; YG8R-UNTR 184.3 ± 3.1, YG8R-omav 126 ± 18, *p < 0.05; Patient 1 257.3 ± 102.3, Patient 1_H_2_O_2_ 315.5 ± 19.9, Patient 1_omav_H_2_O_2_ 123.6 ± 24.1; Patient 2 219.8 ± 44.2, Patient 2_H_2_O_2_ 303.3 ± 17.9, Patient 2_omav_H_2_O_2_ 124.1 ± 13.17; *p < 0.05). The NADH pool decreases combined to an increase of NADH redox state confirmed Complex I inhibition (Bartolomé and Abramov, 2015). Thus, in FRDA models, the activation of the Nrf2 pathway with omav is able to increase the availability of substrates for Complex I and protect the mitochondrial respiration, highlighting its beneficial role in mitochondrial bioenergetics (Dinkova-Kostova et al., 2015; Bartolome et al., 2017).

### Omaveloxolone Prevents Oxidative Stress in Fibroblasts From FRDA Patients

**Figure 2:**
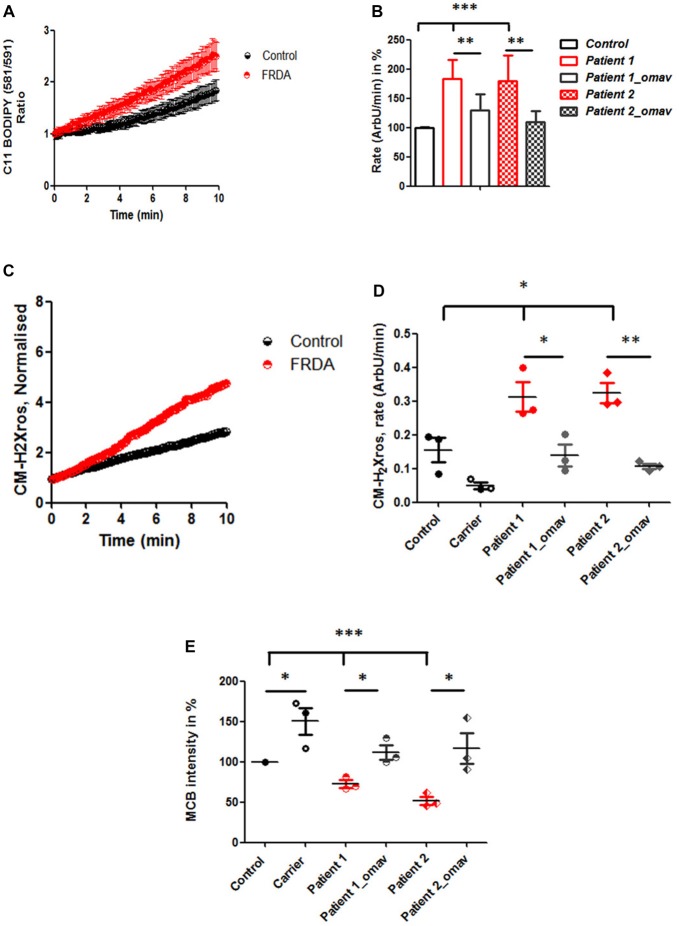
Oxidative stress in FRDA fibroblasts is prevented by omav. **(A)** Lipid peroxidation was measured in Control and FRDA cells with C11 BODIPY (581/591) using live imaging over time. **(B)** The ratio of the fluorophore was calculated (488/562 nm) and analyzed the rate of increase in percentage (%; taken Control as 100%). Patient 1 and 2 fibroblasts untreated showed a significant increase compared to Control (***p < 0.0005) and a significant decrease in rate after omav compared to untreated cells (**p < 0.005). **(C)** The curve shows the kinetic curve of CM-H_2_Xros generated by Control and FRDA cells. **(D)** The rate of increase was expressed in ArbU/min. The rates in untreated patients’ cells were significantly increased compared to Control (*p < 0.05) and patients’ cells untreated and treated with omav showed a significant difference (Patient 1 *p < 0.05; Patient 2 **p < 0.005). **(E)** Glutathione (GSH) was measured with monochlorobimane (MCB) and was lower in patients untreated compared to Control (***p < 0.0005) and the amount seemed to be restored in patients’ cells treated with omav (*p < 0.05).

After confirming that Complex I inhibition is present across FRDA models, we investigated further the omav’s efficacy by assessing FRDA patient’s fibroblasts to confirm that they are a good model for drug testing and potentially recapitulate the disease. One of the markers for oxidative stress in FRDA is lipid peroxidation (Barnham et al., 2004; Abeti et al., 2015, 2016). Therefore, we first assessed the rate of lipid peroxidation in two primary lines of human fibroblasts from FRDA patients (Table 1) using C11 BODIPY (581/591). Patients’ fibroblasts showed a significant increase in lipid peroxidation compared to control, already at steady state (Figures 2A,B). In Figure 2A, we show the increase in fluorescence intensity of C11 BODIPY (581/591) in a mean of experiments from control and FRDA patients. In Figure 2B the histogram represents the rate of the dye in percentage (normalized to control) of patients’ fibroblasts with and without 50 nM omav incubated for 24 h prior experiment. The rate of lipid peroxidation was significantly increased in untreated patients compared to control (Patient 1 183.4 ± 32.8; Patient 2 179.9 ± 43; ***p < 0.0005) and by pre-treating cells with omav the rates decreased significantly in patients (Patient 1_omav 130 ± 27, Patient 2_omav 110 ± 18; **p < 0.005), demonstrating that omav can prevent the pathophysiological increase of lipid peroxidation. We have previously shown that in neurons from an FRDA mouse model, the levels of mROS are also increased, due to lack of frataxin which generates more free radicals and disrupts mitochondrial function (Abeti et al., 2016). We then assessed mROS generation in patient fibroblasts using MitoTracker Red CM-H_2_XRos. As shown in Figures 2C,D, we found that the rate of mROS was increased, and this was successfully prevented by omav (Figure 2D; Control 0.155 ± 0.06, Patient 1 0.3128 ± 0.074; Patient 2 0.3248 ± 0.052; *p < 0.05; Patient 1_omav 0.135 ± 0.055; Patient 1 untreated and treated *p < 0.05; Patient 2_omav 0.107 ± 0.013; Patient 2 untreated and treated **p < 0.05). Interestingly, the carrier sample showed that the rate of mROS was lower than the control. Considering that carriers are asymptomatic but they have about 50% of frataxin levels compared to healthy individuals, it could be possible that a small chronic increase in ROS triggers a constant activation of antioxidant defenses. Indeed, as shown after measuring GSH in these cells, the carrier showed a greater amount compared to control (Figure 2E; 150.2 ± 29.35; *p < 0.05). The level of GSH in patients instead was decreased, but omav was able to restore the levels within the cells (Figure 2E; Patient 1 72.44 ± 8.2; Patient 1_omav 111.6 ± 15.83; *p < 0.05; Patient 2 51.86 ± 8.24, Patient 2_omav 116.7 ± 33.25; *p < 0.05; Patients untreated compared to Control: ***p < 0.0005). These results show that omav is able to increase the antioxidant defenses in FRDA fibroblasts, decrease mitochondrial ROS production and lipid peroxidation, and hence protect against oxidative stress.

### Omaveloxolone Ameliorates Mitochondrial Energy Imbalance

**Figure 3:**
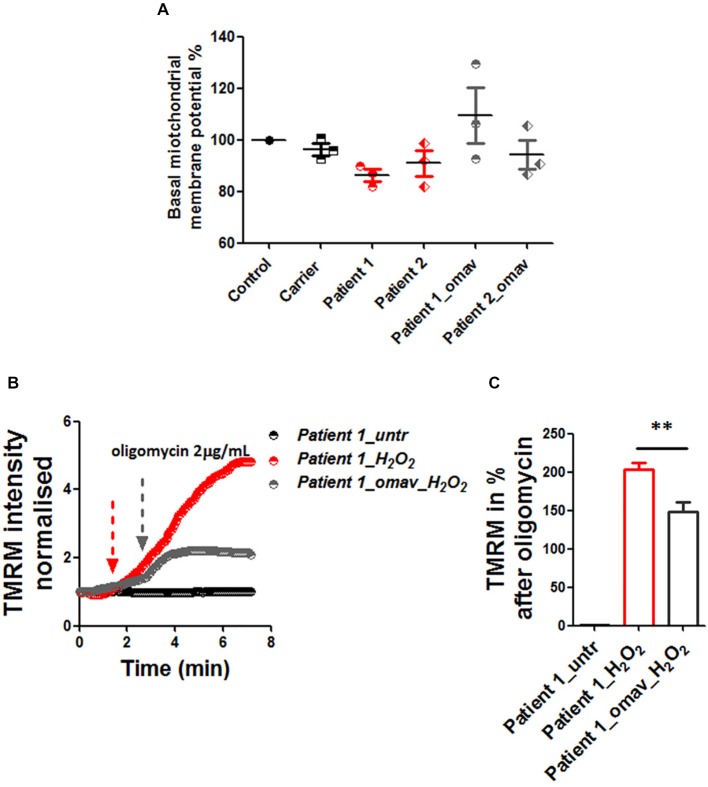
Omav protected ΔΨ_m_ maintenance in stressed fibroblasts. **(A)** Basal ΔΨ_m_ was measured with 25 nM tetramethyl rhodamine, methyl ester (TMRM). Untreated and cells pre-treated with omav did not show significant differences. **(B)** We, therefore, treated patients’ fibroblasts with 1 mM H_2_O_2_ for 2 h and assessed the maintenance of ΔΨ_m_ with 1 μM TMRM (De-quencing mode) and challenged the cells with 2 μg/mL oligomycin. **(C)** The increase of TMRM after oligomycin administration was then calculated in %. Patients’ cells showed a stable line if untreated, however, those treated with H_2_O_2_ showed a marked depolarization which was significantly prevented with omav (**p < 0.005).

The lack of frataxin results in hypersensitivity to oxidative stress and contributes to the mitochondrial pathophysiology. Mitochondrial dysfunction plays an important role in FRDA pathophysiology and given the recently recognized role of Nrf2 in regulating mitochondrial metabolism and function, as reviewed by Esteras et al. (2016) and Holmström et al. (2016), we investigated whether omav could also prevent possible mitochondrial defects in fibroblasts. First we measured the basal levels of ΔΨ_m_ and found that, although there was a trend, patient fibroblasts were not significantly depolarized at steady state compared to controls (Figure 3A; Carrier 96.27 ± 4.09, Patient 1 86.16 ± 3.9, Patient 2 90.90 ± 8.39, Patient 1_omav 109.4 ± 18.69 Patient 2_omav 94.23 ± 9.9; *p* > 0.05). Therefore, we challenged human fibroblasts with 1 mM H_2_O_2_ for 2 h and investigated whether omav could be protective to mitochondrial pathophysiology. Indeed, the presence of H_2_O_2_ disrupted the ability of the electron respiratory chain (ETC) to maintain the ΔΨ_m_ only in patients’ fibroblasts but not in control cells, after administration of oligomycin (Figure 3B). The ΔΨ_m_ was analyzed in de-quench mode, where an increase of fluorescence shows depolarization. Untreated patient cells did not show depolarization, while, H_2_O_2_ treated cells did show a great increase in fluorescence. Interestingly, omav significantly protected the mitochondria from this detrimental effect (Figures 3B,C). Figure 3C summarizes the response to oligomycin in percentage (Patient 1_H_2_O_2_ 203.5 ± 23.06, Patient 1_omav_H_2_O_2_ 148 ± 30.9; **p < 0.005). We can conclude that under induced oxidative stress, FRDA cells show a defect in the maintenance of ΔΨ_m_ but omav protected the cells from the H_2_O_2_ detrimental effect.

### H_2_O_2_ Induced Cell Death Was Prevented by Omaveloxolone

**Figure 4:**
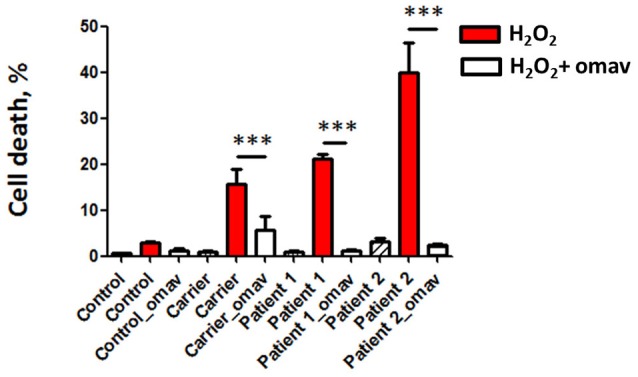
Omav prevents cell death in FRDA fibroblasts. Cells were stained with DAPI for total cells counting and propidium iodide (PI) to visualize dead cells. The % of dead cells was calculated for each case. Hydrogen peroxide (H_2_O_2_) treated cells in red (Carrier, Patients 1 and 2) showed a % of cell death greater than the Control. This was prevented by omav (***p < 0.0005).

FRDA is characterized by atrophy in DRG and cerebellum, which is the result of neuronal death, therefore it is important to determine the efficacy of potential therapeutic drugs preventing cell death. We assessed cell death in patient fibroblasts by PI staining. Patients’ fibroblasts presented hypersensitivity to H_2_O_2_ as shown by higher levels of cell death, which was promptly prevented by omav pre-incubation, confirming the efficacy of the drug (Figure 4; Carrier_H_2_O_2_ 15.67 ± 5.4, Carrier_omav_H_2_O_2_ 5.7 ± 4.3, Patient 1_H_2_O_2_ 21.11 ± 1.428, Patient 1_omav_ H_2_O_2_; Patient 2_H_2_O_2_ 39.95 ± 8.97, Patient 2_omav_ H_2_O_2_ 2.297 ± 0.75; ***p < 0.0005).

## Discussion

**Figure 5:**
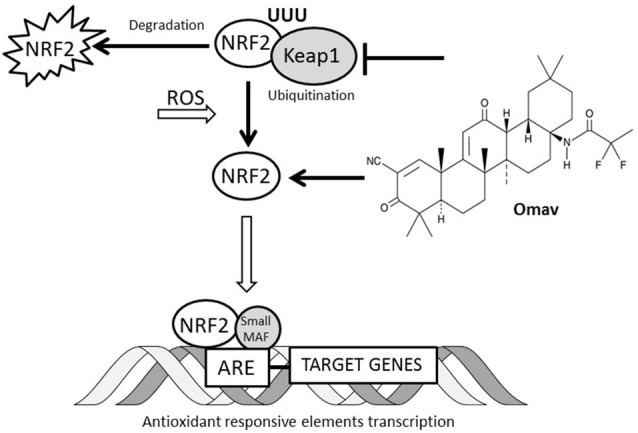
Interaction between omav and Nrf2 pathway. The Nrf2 transcription factor in cells is regulated by ubiquitination and proteosomal degradation through Keap1 protein. The increase of reactive oxygen species (ROS) in cell promotes its translocation to the nucleus where it binds the antioxidant responsive elements (ARE) favoring the transcription of the genes involved in the antioxidant response. Omav is a Nrf2 inducer promotes its activity by inhibiting Keap1 ubiquitinating activity and preventing Nrf2 degradation.

This work sought to understand the potential beneficial effect of omav in CGNs and in fibroblasts from FRDA patients. Mitochondrial dysfunction and oxidative stress are two of the major dysfunctional outcomes shared between neurodegenerative disorders. In particular, in FRDA, both of these detrimental processes contribute significantly to its pathophysiology. Nrf2, a transcription factor which regulates the antioxidant response during oxidative stress, seems to be disrupted, not only in DRG but also in fibroblasts of FRDA models (Paupe et al., 2009; Shan et al., 2013). Moreover, Nrf2 pathway has also an important role in regulating mitochondrial bioenergetics and function (Dinkova-Kostova et al., 2015, 2017). Hence, we have investigated the protective effect of omav, an Nrf2 inducer, in CGNs from the KIKO and YG8R mouse models and FRDA patient fibroblasts. Omav promotes Nrf-2 activity by inhibiting Keap 1 ubiquitinating activity and preventing Nrf2 degradation (Figure 5). By looking at the NADH redox state and pool we found that the pool was low and the redox state was high compared to control. These two parameters demonstrate that there is a lack of mitochondrial substrates for respiration and that Complex I is also inhibited, in all the models tested. Together with our previous experiments on neurons (Abeti et al., 2016), we can conclude that FRDA could be listed on the Complex I deficient diseases. We found that the level of lipid peroxidation and mitochondrial ROS were increased and that GSH was reduced in patient’s fibroblasts at steady state. Previously it has been observed that patient’s fibroblasts fail to activate Nrf2 which consequently reduces the expression of superoxide dismutase 1 and 2 (SOD1/2), Glutathione S-transferase P (GSTP1; crucial for the formation of GSH) and NAD(P)H dehydrogenase (quinone) 1 (NQO1), causing a greater susceptibility to oxidative stress (Paupe et al., 2009). Therefore, we monitored the ability of mitochondria to maintain their membrane potential, stimulating the cells with an oxidant, H_2_O_2_, and found that the maintenance of ΔΨ_m_ was impaired, showing a marked depolarization after oligomycin addition. This implies that the complexes of the ETC were not working properly in FRDA fibroblasts. Disruption in the maintenance of ΔΨ_m_ has been established to be one of the effects of frataxin decrease in neurons and mouse fibroblasts (Abeti et al., 2015, 2016). However, by adding omav for 24 h prior the beginning of the experiments, ΔΨ_m_ was maintained correctly. More importantly, we showed that omav reverses this pathophysiological phenotype, in agreement with previous findings with this compound in other neurodegenerative disorders (Bartolome et al., 2017; Dinkova-Kostova et al., 2017). Oxidative stress and mitochondrial dysfunction are a cause of cell death and we finally studied whether omav could prevent cell death in patients’ fibroblasts. Indeed, omav successfully prevented the death of fibroblasts after an oxidative insult with H_2_O_2_. From our experiments, we can conclude that patients’ fibroblasts present with increase of oxidative stress and this causes greater susceptibility to the administration of oxidants. Omav is effective in counteracting the detrimental effects on the pathophysiology of FRDA, preventing oxidative stress and mitochondrial dysfunction. Moreover, CGNs from FRDA-like mouse models, showing complex I inhibition, were efficiently protected by omav. Previous works on omav demonstrate that this compound triggers the Nrf2 pathway and show that it is cytoprotective (Reisman et al., 2014a,b, 2015; Probst et al., 2015; Rabbani et al., 2018; Table 2). This together with our findings makes omav a very good candidate for treatment in this currently incurable disease.

**Table 2:** Previous and current work on omaveloxolone (omav).

Cellular models	Omav impact on cell functions	References
Mouse and Rat Keratinocytes	NRF2 translocation, m-RNA level of Nrf2, Nqol and glutathione protein expression.	Reisman et al. (2014a,b)
Human Keratinocytes	mRNA expression of many cytoprotective Nrf2 target genes, Protein expression ofNqo1.	Reisman et al. (2015)
Macrophages	Nitrite (NO_2−_) concentrations in the media were measured by Griess reaction and cell viability; m-RNA expression of many cytoprotective Nrf2 target genes (Nqo1, Txnrd1, and Gclc) and m-RNA level of pro-inflammatory genes (Nos2, Ptgs2, cc12, and cc15).	Probst et al. (2015)
Human Tumor cell lines	Check Caspase-3 and caspase-9 cleavage by western blot, m-RNA expression of many cytoprotective Nrf2 target genes (Nqo1, Txnrd1, and Gclc).	Probst et al. (2015)
Diabetic wounds (dorsal mice skin)	Time of diabetic wounds closure, level of ROS with live imaging, level of expression of Nrf2 target antioxidant genes, Nqo1, MnSOD, heme oxygenase 1 (HO-1), glutathione S-transferase (GST), and glutamate cysteineligase (GCL).	Rabbani et al. (2018)
Primary midbrain neurons of PINKI mouse model (Parkinson Disease)	Mitochondrial Membrane Potential, Cell death.	Dinkova-Kostova et al. (2017)
Neuronal-glial co-cultures/Kainic acid induced status epilepticus epilepsy rat model	Level of ROS, Mitochondrial membrane potential, cell death, seizure activity, ATP production.	Shekh-Ahmad et al. (2018)
Human p62 fibroblasts (ALS/FTD), 1362 KD SH-SY5Y cells	Mitochondrial membrane potential, NADH.	Bartolome et al. (2017)
Human FRDA patients’ fibroblasts	Level of ROS, lipid peroxidation, mitochondrial membrane potential, GSH, NADH, cell death.	Current article results
Cerebellar granule neurons (FRDA like mouse models)	NADH	Current article results

## Ethics Statement

This study was carried out in accordance with the recommendations of the NRES Committee London-Harrow, European integrated project on spinocerebellar ataxias (EUROSCA); NHS REC reference: 04/Q0505/21; under which a written informed consent was obtained by the patients for the skin biopsies.

This study was carried out in accordance with the recommendations of Animal (Scientific Procedures) Act 1986 (ASPA). The protocol was approved by the UCL Animal Welfare and Ethical Review Body (AWERB).

## Author Contributions

RA designed the study, conducted and designed experiments, analyzed the results and wrote the manuscript. AB contributed to performing the experiments and edited the manuscript. NE contributed to performing the experiments. PG contributed to designing the study and edited the manuscript.

## Conflict of Interest Statement

The authors declare that the research was conducted in the absence of any commercial or financial relationships that could be construed as a potential conflict of interest.

## Abbreviations

ARE: antioxidant responsive elements
CCD: cooled charge-coupled device camera
DRG: dorsal root ganglia
ETC: electron transport chain
FCCP: Carbonyl cyanide-*4*-(trifluoromethoxy)phenylhydrazone
FRDA: Friedreich’s Ataxia
*FXN*: frataxin gene
GSH: reduced glutathione
GSK3: glycogen synthase kinase 3
GSTP1: Glutathione S-transferase P
H_2_O_2_: hydrogen peroxide
Hrd1: E3 ubiquitin ligase synoviolin
ISCs: iron sulfur clusters
KEAP1: Kelch-like ECH-associated protein 1
MCB: monochlorobimane
mROS: mitochondrial reactive oxygen species
NADH: Nicotinamide Adenine Dinucleotide
NADPH: Nicotinamide Adenine Dinucleotide Phosphate Hydrogen
Nrf2: Nuclear factor (erythroid-derived 2)-like 2
omav: omaveloxolone
PI: propidium iodide
ROS: reactive oxygen species
SOD 2: superoxide dismutase 2
SOD1: superoxide dismutase 1
TMRM: Tetramethyl rhodamine, methyl ester.
